# PKCδ regulates the vascular biology in diabetic atherosclerosis

**DOI:** 10.1186/s12964-023-01361-4

**Published:** 2023-11-16

**Authors:** Peiliang Qin, Changhuai He, Pin Ye, Qin Li, Chuanqi Cai, Yiqing Li

**Affiliations:** Department of Vascular Surgery, Union Hospital, Tongji Medical College, Huazhong University of Science and Technology, Wuhan, 430022 China

**Keywords:** PKCδ pathway, Diabetic atherosclerosis, Vascular biology, Vascular remodeling

## Abstract

Diabetes mellitus, known for its complications, especially vascular complications, is becoming a globally serious social problem. Atherosclerosis has been recognized as a common vascular complication mechanism in diabetes. The diacylglycerol (DAG)–protein kinase C (PKC) pathway plays an important role in atherosclerosis. PKCs can be divided into three subgroups: conventional PKCs (cPKCs), novel PKCs (nPKCs), and atypical PKCs (aPKCs). The aim of this review is to provide a comprehensive overview of the role of the PKCδ pathway, an isoform of nPKC, in regulating the function of endothelial cells, vascular smooth muscle cells, and macrophages in diabetic atherosclerosis. In addition, potential therapeutic targets regarding the PKCδ pathway are summarized.

Video Abstract

**Supplementary Information:**

The online version contains supplementary material available at 10.1186/s12964-023-01361-4.

## Introduction

Diabetes mellitus, characterized by abnormally elevated blood glucose levels, is one of the twenty-first century's fastest growing challenges. According to the International Diabetes Federation (IDF), 1 in 10 adults (age 20–79 years; 537 million individuals) had diabetes in 2021, with the number expected to reach 783 million by 2045 [1]. Patients suffer mostly from chronic complications, including macrovascular and microvascular disease. Macrovascular complications result from lesions to the arteries, leading to large vessel obstructions such as coronary artery disease, atherosclerosis, and peripheral vascular disease [2]. Microvascular complications, characterized by microvascular injuries, include retinopathy, nephropathy, and neuropathy. Atherosclerotic cardiovascular disease (ASCVD), which manifests as coronary heart disease, ischemic stroke, peripheral artery disease, and heart failure, remains the leading cause of death and disability among patients with diabetes mellitus [3]. Hyperglycemia is regarded as the most important factor in the mechanism of diabetic complications, and it has been shown to activate several pathways, including the polyol, nonenzymatic glycation, and advanced glycation end product (AGE) pathways, the production of reactive oxygen species (ROS), and the diacylglycerol (DAG)-protein kinase C (PKC) pathway [2].

**Fig. 1:**
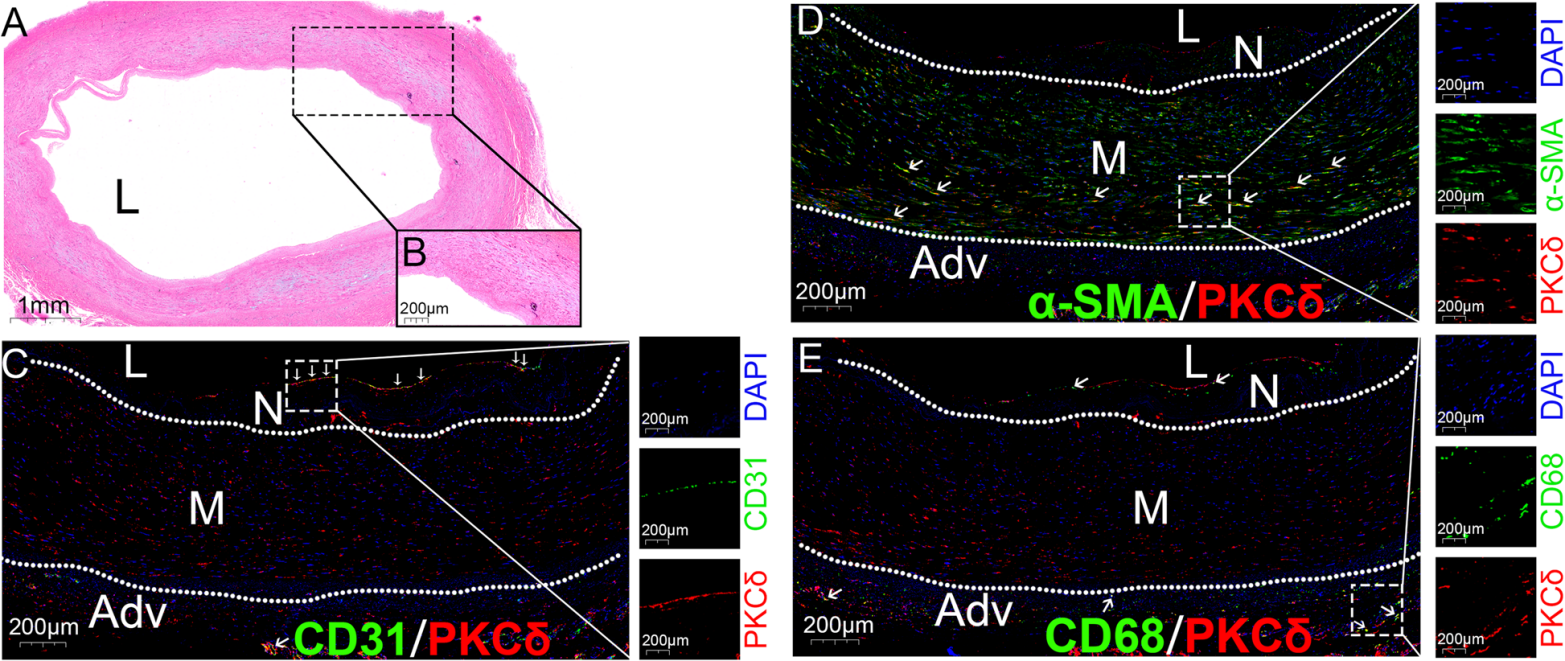
PKCδ, CD31, α-SMA, and CD68 staining in a femoral artery tissue from lower limb of a 60 years diabetes patient who had underwent an amputation surgery (**A**, **B**). Representative images of HE staining of vessels. **C** Positive co-staining of CD31/PKCδ was observed in intimal layer. CD31 shown in green, PKCδ in red, and DAPI in blue. **D** Positive co-staining of α-SMA/PKCδ was observed in media layer. α-SMA shown in green, PKCδ in red, and DAPI in blue. **E** Positive co-staining of CD68/PKCδ was observed in neointimal and adventitial layers. CD68 shown in green, PKCδ in red, and DAPI in blue. The magnification scale of HE image was 5X. Arrows show positive colocalized staining. L, lumen; M, media; N, neointima; Adv, adventitia

The PKCs are a family of serine/threonine-related protein kinases that play indispensable roles in several signal transduction pathways and cellular functions [2]. PKCδ is a PKC isoform belonging to the novel PKC (nPKC) subgroup that is Ca^2+^-independent and phospholipid- and DAG-activated [4]. PKCδ was found to be activated in a number of atherosclerotic cardiovascular diseases as well as diabetic complications, indicating that it may be a mediator of diabetes-related atherosclerosis. Atherosclerosis is a complex process involving various types of cells, including endothelial cells (ECs), vascular smooth muscle cells (VSMCs), monocytes/macrophages, and so on. To determine the expression of PKCδ in ECs, VSMCs, and macrophages in human vessels, we stained paraffin sections of a vessel from the amputated limb of a male diabetes patient, with his informed consent (Fig. 1). He experienced pain at rest due to severe arterial atherosclerotic occlusions in the left lower extremity and amputation was indicated. The patient was well informed, and several vessels were collected after amputation. The staining was from a non-occluded artery with thin neointima. Markers of ECs (CD31), VSMCs (α-SMA), and macrophages (CD68) were stained green and the marker of PKCδ was stained red. Although the functions of PKCδ have been discussed in previous reviews, they have not been reviewed in detail [5, 6]. In this review, we summarize the role of PKCδ in regulating the dysfunction of endothelial cells, vascular smooth muscle cells, and monocytes/macrophages in non-DM and DM conditions to provide a comprehensive understanding of the role of PKCδ in diabetic atherosclerosis.

## PKCδ in the dysfunction of endothelial cells

Endothelial cells dysfunction leads to the earliest detectable changes, such as focal permeation, trapping, and physicochemical modification of circulating lipoprotein particles in the sub-endothelial space, and plays a vital role in the pathophysiology of atherosclerosis. Endothelial dysfunction is characterized by impaired endothelium-dependent vasodilation, hyperpermeability, leukocytes adhesion, chronic inflammation, heightened oxidative stress, endothelial-to-mesenchymal transition, and endothelial cells senescence and apoptosis [7].

Healthy endothelium regulates vascular tone and structure and protects vessels from thrombosis [8]. Impaired vascular tone can lead to increased endothelial permeability, platelet aggregation, leukocytes adhesion, and the generation of cytokines. Hyperpermeability can be induced by a variety of cytokines, including vascular endothelial growth factor (VEGF), histamine, and thrombin, as well as other factors, such as high levels of oxidative stress and inflammation [7]. Damage to endothelial barrier integrity leads to lower NO availability, vascular swelling/edema, and abnormal hemostasis. Under pathologic conditions, the expression of adhesion molecules such as VCAM-1, ICAM-1, E-selectin, and MCP-1 is induced by proinflammatory mediators. These adhesion molecules enhance leukocytes adhesion and transmigration while also triggering inflammation, which is at the core of atherosclerosis. Furthermore, heightened oxidative stress facilitates the formation of ox-LDL, activates endothelial cells, upregulates adhesion molecule expression, alters vascular tone, and leads to EC apoptosis [9, 10]. Endothelial-to-mesenchymal (EndoMT) transition is associated with tissue remodeling, inflammation, disturbed blood flow, and plaque formation. Notably, EndoMT-derived fibroblasts show an unstable plaque phenotype, which increases the risk of plaque rupture. Apoptosis of endothelial cells is also linked to the development of atherosclerotic plaque, particularly plaque rupture, possibly by secreting apoptosis-induced extracellular vesicles [11]. Endothelial cells senescence contributes to atherosclerosis by regulating the levels of endothelin-1 (ET-1), monocyte chemoattractant protein-1 (MCP-1), angiotensin II, and nitric oxide (NO) [12]. These pathophysiologic events interact with one another, accelerating the development of atherosclerosis.

**Fig. 2:**
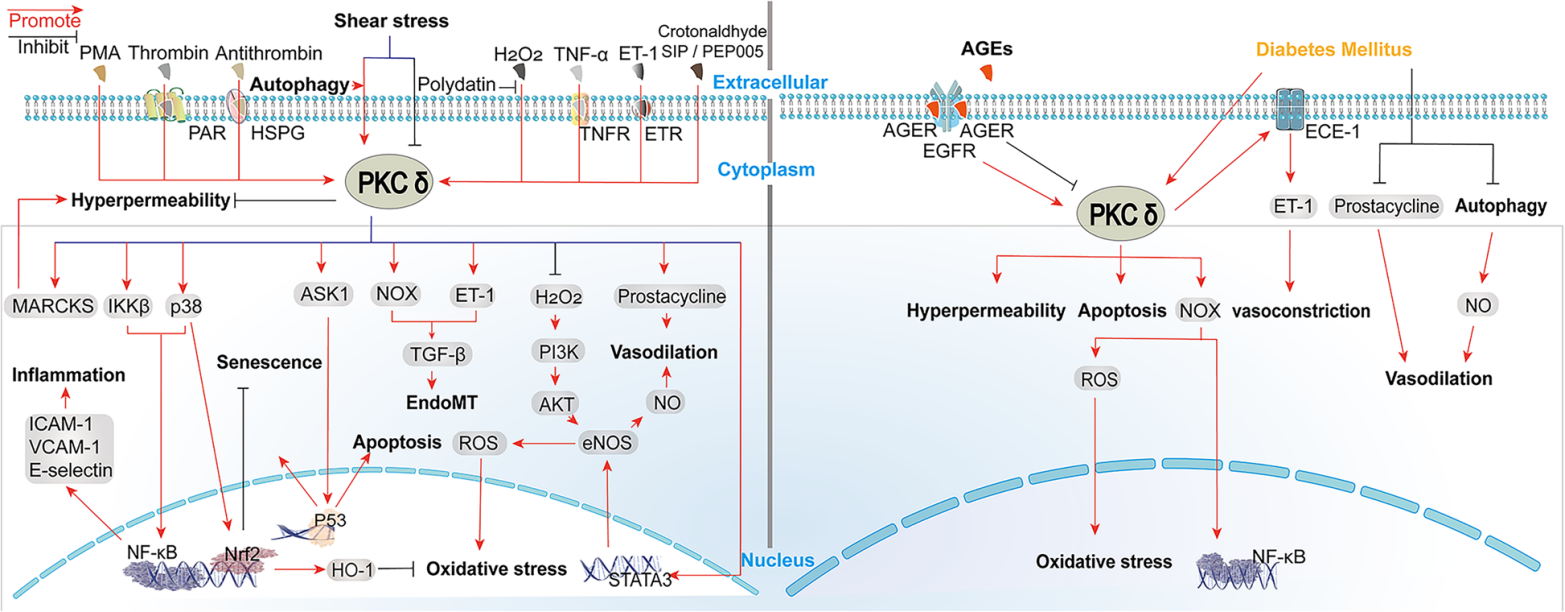
PKCδ-mediated signal transduction pathways in endothelial cells in non-DM studies (left) and DM studies (right)

In this section, we summarize the role of PKCδ in mediating endothelial dysfunction in non-DM and DM conditions. Figure 2 shows the potential cell signaling pathways. In addition, Table 1 summarizes the results of animal experiments. The effects of PKCδ knockout/knockdown (KO/KD) on endothelial function were investigated in both non-DM and DM models. In addition, changes in endothelial function in DM animals are listed.

**Table 1:** Animal studies indicating the role of PKCδ in regulating the function of endothelial cells

Pathophysiological process		Group 1	Group 2 (vs. group 1)	Group 3 (vs. group 1)	Group 4 (vs. group 3)
	Rodents with DM	-	-	+	+
	PKCδ KO/KD rodents	-	+	-	+
Endothelial PKCδ expression		Base	Decrease	Increase [13]	Decrease
Vasodilation (tissue)		Base	Not mentioned	Decrease [13]	Increase [13]
Vasoconstriction (tissue)		Base	Not mentioned	Increase [14]	Decrease [14]
Inflammatory cell infiltration		Base	Decrease [15] Increase [16]	Not mentioned	Not mentioned
Neutrophils adhesion		Base	Decrease [17]	Not mentioned	Not mentioned
Neutrophils migration		Base	Decrease [17]	Not mentioned	Not mentioned
Tight junction protein expression		Base	Increase [18]	Decrease [19]	Increase [19]
Hyperpermeability		Base	Decrease [18] Increase [16]	Increase [19]	Decrease [19]

Group 1, normal animals; Group 2, PKCδ knockout/knockdown animals without DM; Group 3, DM animals; Group 4, PKCδ knockout/knockdown animals with DM

### PKCδ regulates endothelium-dependent vasodilation and vasoconstriction

The endothelium plays an important role in the regulation of vascular tone. For one thing, the endothelium produces a series of vasodilators, including nitric oxide (NO), hydrogen sulfide (H_2_S), carbon monoxide, arachidonic acid metabolites, and H_2_O_2_ [7]. The endothelium also generates several vasoconstrictor molecules, such as endothelin 1 (ET-1), angiotensin II (Ang-II), thromboxane A2 (TxA2), thrombin, superoxide anion, and other contracting factors [7]. PKCδ has been reported to participate in the production of both vasodilators and vasoconstrictors.

NO, produced through the endothelial isoform of nitric oxide synthase (eNOS), regulates vascular tone and maintains the anti-thrombogenic characteristics of the vascular wall [20]. Diabetes is associated with an impairment in the production or bioavailability of NO, which can accelerate the formation of atherosclerotic lesions. The exact role of PKCδ in regulating NO generation is still under debate; it has been reported to be a promoter of NO production. Diabetes is also associated with coagulation abnormalities and thrombin activation, and the inhibition of thrombin ameliorates endothelial dysfunction. Motley et al. [21] reported that PKCδ plays an indispensable role in thrombin-induced Ser1179 phosphorylation-dependent eNOS activation and NO production in bovine aortic endothelial cells. Moreover, evidence has shown that inadequate autophagy in endothelial cells from patients with diabetes impairs NO signaling [22]. PKCδ T505 activation restores shear stress-induced eNOS S1177 phosphorylation and promotes NO production associated with impaired autophagy [23]. On the other hand, PKCδ also seems to play a negative role. Kumar et al. [24] argued that PKCδ activity was restrained under shear stress, leading to H_2_O_2_/PI3K/Akt activation, eNOS phosphorylation, and increased NO production in pulmonary arterial endothelial cells. Sud and Black [25] added that increased ET-1 signaling activated PKCδ and enhanced NO production. Notably, PKCδ-mediated STATA3 activation was found to take part in both ET-1-suppressed and shear stress-induced eNOS expression and NO generation in fetal pulmonary artery endothelial cells [25, 26]. Furthermore, prostacyclin, another vasodilator, was reduced in human aortic endothelial cells with induced hyperglycemia [27]. Panicker et al. demonstrated that PKCδ was required for antithrombin-induced prostacyclin expression in endothelial cells [28]. Moreover, PKCδ inhibition in rat aorta with STZ-induced DM restored endothelium-dependent dilation, indicating a deleterious role of PKCδ in the dysfunction of endothelium-dependent dilation [13].

The expression of ET-1, a potent vasoconstrictor, was enhanced under hyperglycemia via the activation of endothelin converting enzyme-1 (ECE-1) in human umbilical vein endothelial cells (HUVECs), partly due to the activation of PKCδ [29]. Additionally, increased ET-1 in bovine retinal pericytes and capillary retinal endothelial cells under high-glucose conditions was also partially mediated by PKCδ, which exacerbated the ischemic state in the retina [30]. Furthermore, prostaglandin E_2_ (PGE_2_), an important and ubiquitous vasoactive eicosanoid, was shown to be a vasoconstrictor in some vessels, including rat mesenteric artery. Enhanced EP3 receptor-mediated vasocontraction in mesenteric arteries from Goto-Kakizaki rats with type 2 diabetes resulted from PKCδ activation [14].

### PKCδ regulates hyperpermeability

The vascular endothelium acts as a semipermeable barrier between vascular smooth muscle cells and the vascular lumen, and hyperpermeability leads to impaired vascular homeostasis [7]. Gaudreault et al. [31] reported an increase in PKC βII and a decrease in PKCδ expression in coronary endothelial cells of diabetic rats, contributing to endothelial hyperpermeability and coronary dysfunction. Kim et al. [32] also documented a protective role of PKCδ and a deleterious role of PKC βII in maintaining the blood–brain barrier during aglycemic hypoxia. Conversely, Kim et al. [19] argued that activation of PKCδ, which is related to its subcellular translocation, leads to increased vascular permeability in response to diabetes and the PKCδ inhibition restores the loss of tight junction proteins in retinal vessels. PKCδ inhibition was also shown to significantly reduce TNF-α-mediated hyperpermeability, decrease transendothelial electrical resistance (TEER), and interrupt tight junction expression in vitro in activated HBMVECs and rat brain in vivo 24 h after cecal ligation and puncture (CLP) induced sepsis [18]. PKCδ also appears to be required for phorbol 12-myristate 13-acetate (PMA)- and diacylglycerol (DAG)-induced myristoylated alanine-rich C-kinase substrate (MARCKS) phosphorylation and hyperpermeability in pulmonary microvascular endothelial cells and thrombin-induced loss of human pulmonary artery endothelial cells barrier integrity [33, 34].

### PKCδ regulates leukocytes adhesion and transmigration

Increased leukocytes adhesion, rolling, and transmigration into the subendothelial space is an important cause of endothelial dysfunction, which is attenuated by several risk factors, including hyperglycemia, and is often associated with chronic inflammation [7]. Several studies describe different functions of PKCδ in mediating inflammatory cytokine-induced neutrophils adhesion and transmigration. Mondrinos et al. [15] demonstrated that PKCδ inhibition in pulmonary microvascular endothelial cells (PMVECs) decreased IL-1β-mediated neutrophils transmigration. PKCδ inhibition also reduced TNF-α-mediated neutrophils adhesion and migration across human brain microvascular endothelial cells (HBMVECs) [18]. In vivo studies also proved that intratracheal administration of δ-PKC TAT peptide significantly attenuated inflammatory cell infiltration and concomitant endothelial ICAM-1 and VCAM-1 expression in a rat model of sepsis-induced indirect pulmonary injury [15]. However, Ahn et al. reported acquired enhanced neutrophils transmigration in PKCδ knockout mice and higher permeability in an LPS-induced acute lung injury model [16].

Furthermore, PKCδ regulates leukocytes adhesion and transmigration by regulating signal pathways in leukocytes. PKCδ was reported to mediate the phosphorylation of MARCKS, promoting the migration and adhesion of neutrophils in vitro [35]. Bone marrow neutrophils isolated from wild-type mice showed significant adhesion and migration across endothelial cells in vitro compared to those from PKCδY155F knock-in mice [17]. In vivo studies also illustrated the important role of PKCδ tyrosine 155 phosphorylation in neutrophils migration into the lungs of septic mice [17].

### PKCδ regulates endothelium-mediated inflammation

Diabetes is associated with chronic inflammation, which is mainly due to increased plasma concentrations of C-reactive protein (CRP), fibrinogen, interleukin-6 (IL-6), interleukin-1 (IL-1), and TNF α [36]. These inflammatory cytokines increase vascular permeability, alter vasoregulatory responses, promote leukocytes adhesion to endothelium, facilitate thrombus formation, inhibit anticoagulant pathways, and impair fibrinolysis function. The mechanism involves the regulation of several factors, including endothelial intercellular adhesion molecule-1 (ICAM-1), vascular adhesion molecule-1 (VCAM-1), E-selectin, MCP-1, NO, prostacyclin, ET-1, interleukin-8 (IL-8), and plasminogen activator inhibitor-1 (PAI-1). PKCδ was reported to regulate thrombin-induced ICAM-1 gene transcription by a dual mechanism involving activation of IKKβ, which mediates NF-κB binding to the ICAM-1 promoter, and p38 MAP kinase, which enhances the transactivation potential of the bound NF-κB p65 [37]. Notably, the PKCδ/NF-κB signaling pathway also participates in thrombin-induced VCAM-1 expression [38]. In addition, TNF-α upregulates ICAM-1 and VCAM-1 expression, which is inhibited by antithrombin via a PKCδ-dependent mechanism [28]. Histamine-mediated activation of PKCδ increases the expression of VCAM-1, ICAM-1, and E-selectin synergistically in HUVECs in response to a secondary stimulus of sphingosine 1-phosphate (S1P) [39]. PKCδ also mediates endothelial E-selectin and ICAM-1 induction, IL-8 expression, and leukocytes recruitment when exposed to PEP005, an anti-tumor agent [40].

### PKCδ regulates oxidative stress

Oxidative stress is a state of imbalance resulting from the increased generation of reactive oxygen species (ROS) and/or a weakened antioxidant system [41]. ROS, mainly derived from xanthine oxidase, NADPH oxidases (NOX), uncoupled eNOS, and dysfunctional mitochondria in endothelial cells, play an important role in the progression of atherosclerosis. Diabetes is a potent oxidative stress inducer. It has been established that NADPH oxidase-dependent ROS generation and NF-κB activation are upregulated in endothelial cells, which is induced by advanced glycated end products (AGEs) [42]. Notably, induction by AGEs is protected by advanced glycated end-product receptor 1 (AGER1) via EGFR/PKCδ pathway inhibition [42]. Furthermore, uncontrolled eNOS activity mediated by PKCδ activation or ɛPKC inhibition also leads to ROS and reactive nitrogen species (RNS) formation in endothelial dysfunction [43]. Polydatin, which is extracted from the root stem of a traditional Chinese herbal medicine, *Polygonum cuspidatum Sieb*, attenuated H_2_O_2_-induced phosphorylation of PKCδ and protected HUVECs against oxidative stress injury [44]. PKCδ was also found to be involved in antioxidant pathways. Lee et al. [45] reported that crotonaldehyde-induced heme oxygenase-1 (HO-1) expression is mediated by the PKCδ–p38 MAPK–Nrf2–HO-1 pathway in HUVECs, which is an adaptive response to oxidative stress.

### PKCδ regulates endothelial-to-mesenchymal transition

The endothelial-to-mesenchymal transition (EndoMT), in which endothelial cells lose their endothelial characteristics and acquire a mesenchymal-like morphology and gene expression pattern, is another cause of endothelial dysfunction and atherosclerosis [7]. Considered to be the main driver of EndoMT, TGF-β has been demonstrated to be activated in diabetic endothelial cells through several signaling pathways, including ET-1, PAI-1, Ang-II, and NOX [46]. As mentioned above, PKCδ mediates the activation of ET-1 and NOX, which subsequently facilitates the expression of TGF-β. It has also been reported that PKCδ and c-Abl are necessary for TGF-β-induced EndoMT [47]. Furthermore, PKCδ may promote the activity of protein phosphatase 2a (PP2A), which contributes to EndoMT [46].

### PKCδ regulates endothelial cells senescence and apoptosis

Endothelial senescence and apoptosis can both be induced by diabetes or high glucose, leading to vascular dysfunction and atherosclerosis [7]. It has been established that PKCδ is involved in high-glucose-induced apoptosis in HUVECs [48]. PKCδ mediates diabetes-induced oxidative stress, which is associated with endothelial senescence and cell death. Nuclear factor-erythroid 2-related factor 2 (Nrf2) is an important regulator of antioxidant expression and prevents cell senescence. PKCδ is required for Nrf2 serine 40 phosphorylation, antioxidant induction of defensive gene expression, and promoting cell survival [49]. Notably, PKCδ-activated mTOR also interacts with Nrf2 and delays endothelial senescence. P53, which is activated in human endothelial cells exposed to high glucose, was recognized as an important factor promoting cell senescence and apoptosis. It was proved that PKCδ promotes the accumulation of p53 and apoptosis in H_2_O_2_-treated bovine aortic endothelial cells (BAECs) [50]. Furthermore, apoptosis signal-regulating kinase 1 (ASK1)-induced cellular senescence may be mediated through the p53-dependent signaling pathway, and PKCδ may be an ASK1 inducer [51, 52]. In addition, except for its vasodilation function, NO was also demonstrated to reduce endothelial senescence, and PKCδ may aggravate cell senescence by attenuating NO production [53].

## PKCδ in the dysfunction of vascular smooth muscle cells

There are few intimal VSMCs in normal arteries, and they have a low turnover. VSMCs migrate from the media to the intima during atherosclerotic plaque formation, where they accumulate, proliferate, and produce extracellular matrix, which is the main component of atherosclerotic plaque [54]. A fibrous cap composed of smooth muscle cells and interstitial collagen fibers surrounds the necrotic core of an atherosclerotic plaque. The loss of smooth muscle cells due to apoptosis, proptosis, and necrosis can result in a thinner fibrous cap, increasing the likelihood of plaque rupture and atherothrombotic events [55]. Enhanced migration, proliferation, and apoptosis of smooth muscle cells hasten the formation and rupture of atherosclerotic plaque.

**Fig. 3:**
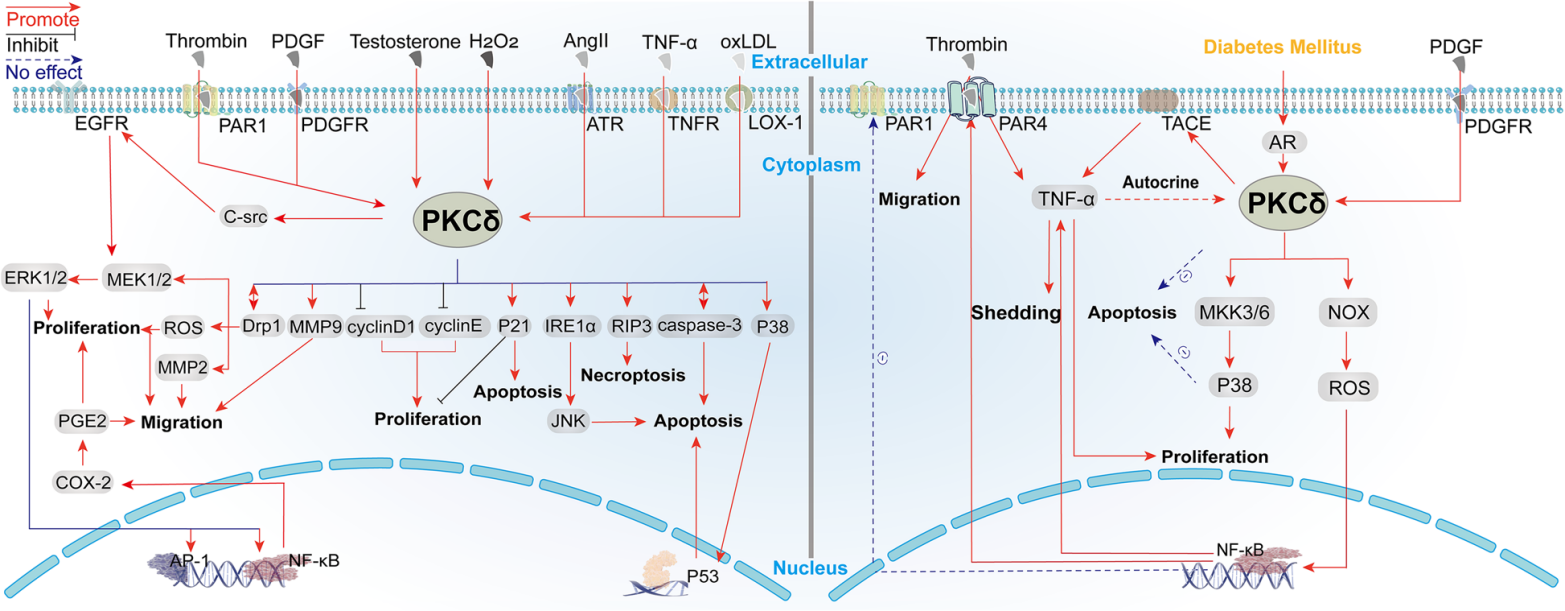
PKCδ-mediated signal transduction pathways in smooth muscle cells in non-DM studies (left) and DM studies (right)

In this section, we also review the role of PKCδ in mediating VSMCs dysfunction in non-DM and DM conditions. Figure 3 summarizes the reported cell signaling pathways. In addition, Table 2 summarizes the results of animal experiments. The effects of PKCδ knockout/knockdown (KO/KD) on the function of VSMCs are shown in both non-DM and DM models. In addition, the changes in VSMCs function in DM animals are also listed.

**Table 2:** Animal studies indicating the role of PKCδ in regulating the function of vascular smooth muscle cells

Pathophysiological process		Group 1	Group 2 (vs. group 1)	Group 3 (vs. group 1)	Group 4 (vs. group 3)
	Rodents with DM	-	-	+	+
	PKC δ KO/KD rodents	-	+	-	+
VSMCs PKC δ expression		Base	Decrease	Increase [56]	Decrease
VSMCs number in atherosclerotic lesions		Base	Increase [57]	Not mentioned	Not mentioned
VSMCs proliferation		Base	Decrease [58]	Increase [56]	Not mentioned
Mitogen-stimulated VSMCs proliferation		Base	No change [57]	Not mentioned	Not mentioned
PDGF-induced VSMCs proliferation		Base	Not mentioned	Increase [56]	Not mentioned
VSMCs adhesion		Base	Decrease [59]	Not mentioned	Not mentioned
Mechanical stress-induced VSMCs migration		Base	Decrease [60]	Not mentioned	Not mentioned
PDGF-induced VSMCs migration		Base	Decrease [59]	Increase [56]	Not mentioned
VSMCs chemotaxis		Base	Decrease [58]	Not mentioned	Not mentioned
PDGF-induced VSMCs apoptosis		Base	Not mentioned	No change [56]	Not mentioned
VSMCs death in atherosclerotic lesions		Base	Decrease [57]	Not mentioned	Not mentioned
Arterial injury-induced VSMCs apoptosis		Base	Decrease [61]	Not mentioned	Not mentioned
Oxidative-induced VSMCs death		Base	Decrease [62]	Not mentioned	Not mentioned

Group 1, normal animals; Group 2, PKCδ knockout/knockdown animals without DM

Group 3, DM animals; Group 4, PKCδ knockout/knockdown animals with DM

### PKCδ regulates the migration and proliferation of VSMCs

The exact role of PKCδ in regulating VSMCs migration and proliferation remains undefined. Some researchers regard PKCδ as an inhibitor. In non-DM studies, Fukumoto et al. [63] reported that PKCδ inhibited VSMCs proliferation by arresting cells in G1 mainly by inhibiting the expression of cyclins D1 and E. Bowles et al. [64] also proved the involvement of PKCδ-mediated p21^cip1^ upregulation and cyclin D1 and E downregulation in the antiproliferative and pro-apoptotic effects of testosterone on coronary smooth muscle cells (CSMCs). Notably, evidence has shown that aromatization of testosterone to estrogen is not necessary for PKCδ-mediated inhibition of CSMCs proliferation by testosterone [64]. Some researchers have reported contrary results. Lim et al. [65] found that in response to atherosclerotic stimulus, dynamin-related protein 1 (Drp1), a critical molecule regulating mitochondrial fission, and PKCδ, showed reciprocal activation. For one thing, Drp1 enhanced MEK1/2-ERK1/2 signaling cascade, MMP2, and ROS, which promoted VSMCs proliferation and migration, and the promotion was abolished by mitochondrial division inhibitor (Mdivi-1). In addition, PKCδ facilitated VSMCs migration by activating MMP9 independent of Drp1. PGE2 has been considered to be a promoter of VSMCs proliferation and migration. Cyclooxygenase 2 (COX-2), a rate-limiting enzyme in the synthesis of prostaglandins (PGs), including PGE2, is not detectable in most normal tissues but can be induced by thrombin in VSMCs [66]. Hsieh et al. revealed that thrombin-induced COX 2 activation was mediated through PKCδ /c-Src-dependent EGFR transactivation, MEK-ERK1/2, AP-1, and NF-κB. Platelet-derived growth factor (PDGF), an important factor promoting atherosclerosis, is known as a regulator of the proliferation and migration of VSMCs. PDGF induces the translocation of PKCδ from the cytosol to the post-nuclear particulate fraction, which is inhibited by TGF-beta1 [67]. Evidence has shown that PKCδ mediates PDGF-induced ERK1/2 activation, regulating the proliferation and migration of VSMCs [58, 68]. Interestingly, overexpression of PKCδ inhibited ERK1/2 activity, leading to decreased proliferation and migration of VSMCs, while VSMCs isolated from PKCδ knockout mice showed diminished chemotaxis and proliferation compared with VSMCs from PKCδ^+/+^ mice, revealing a complex role of PKCδ in regulating VSMCs proliferation and migration. Furthermore, animal studies also demonstrated that PKCδ activation is necessary for the adhesion of VSMCs, which contributes to their migration [59]. Li et al. [60] noted that mechanical stress activates PKCδ translocation to the cytoskeleton, which is related to decreased VSMCs migration.

However, DM studies show that PKCδ is inclined to be a promoter of VSMCs proliferation and migration. As previously indicated, the polyol pathway, which reduces glucose to sorbitol and then oxidizes sorbitol to fructose, is activated under hyperglycemia [69]. Aldose reductase (AR) is the catalyst of the rate-limiting step. Enhancement of the polyol pathway leads to changes in cell osmolarity and redox state and causes subsequent tissue injury, and AR inhibition relieves or even reverses diabetic lesions in the lens, kidney, and nerves. At the same time, inflammatory cytokines such as tumor necrosis factor alpha (TNF-α) are increased in various tissues in diabetes, which contributes to insulin resistance and reflects the severity of the disease [70]. High-glucose-induced TNF-α expression and vascular smooth muscle cells growth are mediated by the AR/PKCδ/NADPH oxidase/NF-κB pathway. Notably, the increased TNF-α causes autocrine stimulation of PKCδ, which appears to be an essential mediator of HG-induced VSMCs growth. Furthermore, aldose reductase regulates hyperglycemia-induced ectodomain shedding of TNF-α through the PKCδ/TNF-alpha converting enzyme (TACE) pathway [71]. Thrombin, well known as a key component of the coagulation cascade, also facilitates the proliferation and migration of vascular smooth muscle cells [72]. It is involved in the activation of a unique family of G protein-coupled receptors, the protease-activated receptors (PARs). In diabetes mellitus, the isoform PAR-4 rather than PAR-1 is activated through a PKCδ/NF-κB-dependent pathway, which enhances VSMCs migration and TNF-α expression. Moreover, PKCδ was found to be further enhanced in PDGF-BB-induced VSMCs from diabetic rats, promoting subsequent p38 phosphorylation via MAPK kinase (MKK) 3/6, facilitating VSMC proliferation and migration, increasing the cyclooxygenase-2 level, and inducing arachidonic acid release but not apoptosis [56].

### PKCδ regulates VSMCs apoptosis

PKCδ is generally accepted to be a pro-apoptotic molecule involved in several diabetic complications [73–75]. In one study, overexpression of PKCδ was sufficient to induce apoptosis, while its suppression eliminated H_2_O_2_-induced apoptosis in A10 VSMCs [76]. PKCδ also participates in oxidized LDL-induced ER stress-mediated apoptosis mainly through the IRE1α/JNK pathway in VSMCs [77]. P38 MAPK, a subtype of conventional MAPKs that works in a typical three-tiered module, mediates pro-apoptotic processes through transcriptional and/or post-transcriptional regulation in cells exposed to extracellular or intracellular stress [78]. It has been shown to be activated in smooth muscle cells in both a PKCδ-dependent and a PKCδ-independent way in high glucose or diabetes [79]. P38 MAPK is required in the accumulation and phosphorylation of p53 in VSMCs, which is stimulated by PKCδ [76]. Notably, phosphorylation of p53 on Ser(46) by PKCδ was also found to lead to an apoptotic response to DNA damage [80]. Moreover, caspase-3-mediated PKCδ cleavage is necessary for VSMCs apoptosis induced by oxidative stress [81]. Interestingly, PKCδ inhibition diminishes caspase-3 activation and PKCδ cleavage, indicating that PKCδ acts both upstream and downstream of caspase-3. PKCδ also contributes to TNF-α-induced VSMCs necroptosis by regulating RIP3 expression [82]. However, PKCδ upregulation showed no significant effect on serum withdrawal-induced apoptosis under hyperglycemia [83]. In animal studies, Yamanouchi et al. [61] put forward that PKCδ mediates arterial injury-induced VSMCs apoptosis, alleviating intimal hyperplasia. Leitges et al. [57] reported a higher number of VSMCs in arteriosclerotic lesions of PKCδ^−/−^ mice compared to wild-type animals, which was related to decreased VSMCs death in PKCδ^−/−^ mice. Furthermore, VSMCs isolated from aortas of PKCδ^−/−^ mice showed resistance to several pro-apoptotic stimuli, manifested as decreased caspase-3 activation, poly (ADP-ribose) polymerase cleavage, and cytochrome c release, compared with VSMCs from wild-type mice [57]. Notably, nuclear magnetic resonance spectroscopy showed elevated cellular glutathione levels in PKCδ^−/−^ VSMCs, which leads to resistance to cell death induced by oxidative stress [62].

## PKCδ in monocytes and macrophages dysfunction

In the formation of atherosclerotic plaques, classic monocytes/macrophages play pro-inflammatory roles [54]. They are activated by pathogen-associated molecular patterns (PAMPs) such as lipopolysaccharide and damage-associated molecular patterns (DAMPs) such as ox-LDL in the environment of the arterial wall, initiating an inflammatory response. Local inflammation induces the expression of several inflammatory factors, including MCP-1, which plays a major role in recruiting circulating monocytes, which attach to endothelial cells before migrating into the intima. Once in the intima, monocytes mature into macrophages and express scavenger receptors to bind lipoproteins and become foam cells. Studies on the consequences of macrophages death in atherosclerosis revealed opposing roles for macrophages apoptosis in plaque formation [84]. In early lesions, macrophages apoptosis limits lesion cellularity and suppresses plaque progression, while in advanced lesions, macrophages apoptosis increases the possibility of plaque disruption and acute luminal thrombosis. The dysfunction of monocytes/macrophages aggravates inflammation, increases monocytes adhesion, transmigration, and differentiation, and promotes foam cell formation, eventually accelerating the progression of atherosclerosis.

**Fig. 4:**
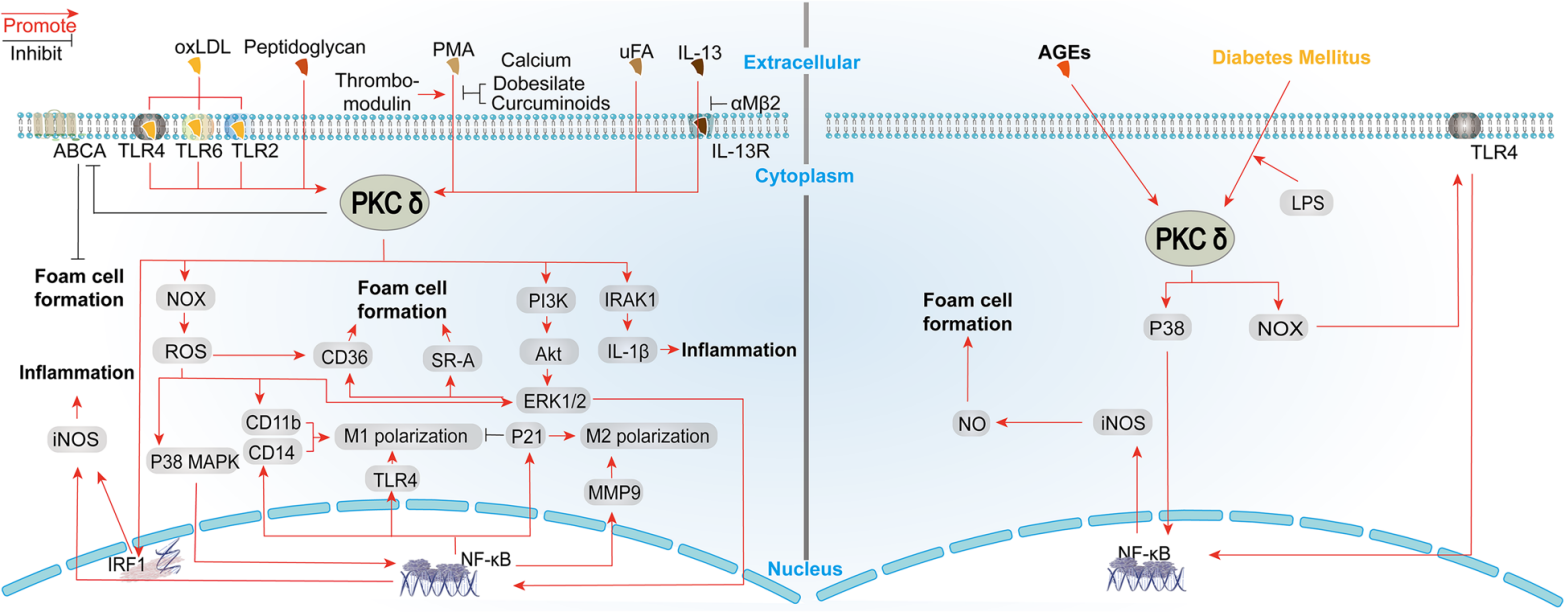
PKCδ-mediated signal transduction pathways in monocytes/macrophages in non-DM studies (left) and DM studies (right)

In this section, we summarize the role of PKCδ in mediating monocytes/macrophages dysfunction in non-DM and DM conditions. Figure 4 shows the potential cell signaling pathways. In addition, Table 3 summarizes the results of animal experiments. The effect of PKCδ knockout/knockdown (KO/KD) on monocytes/macrophages function was investigated in both non-DM and DM models. In addition, changes in monocytes/macrophages function in DM animals are listed.

**Table 3:** Animal studies indicating the role of PKCδ in regulating the function of monocytes/macrophages

Pathophysiological process		Group 1	Group 2 (vs. group 1)	Group 3 (vs. group 1)	Group 4 (vs. group 3)
	Rodents with DM	-	-	+	+
	PKC δ KO/KD rodents	-	+	-	+
Macrophages PKCδ expression		Base	Decrease	Increase [85]	Decrease
Macrophages proinflammatory biomarker expression		Base	Not mentioned	Not mentioned	Decrease [86]
Macrophages uptake of oxLDL		Base	No change [87]	Not mentioned	Not mentioned
Foam cell formation		Base	No change [87]	Not mentioned	Not mentioned
Atherosclerotic lesions		Base	Increase [85]	Not mentioned	Increase [85]
Splenomegaly		Base	Increase [85]	Not mentioned	Increase [85]
Macrophages number in aortic plaque/spleen		Base	Increase [85]	Not mentioned	Increase [85]
Macrophages apoptosis in aortic plaque/spleen		Base	Decrease [85]	Not mentioned	Decrease [85]
Macrophages proliferation in aortic plaque/spleen		Base	Increase [85]	Not mentioned	Increase [85]
Monocytes uptake into arterial wall		Base	No change [85]	Not mentioned	No change [85]
Inflammatory cytokines expression in aortic plaque		Base	Increase [85]	Not mentioned	Increase [85]

Group 1, normal animals; Group 2, PKCδ knockout/knockdown animals without DM

Group 3, DM animals; Group 4, PKCδ knockout/knockdown animals with DM

### PKCδ regulates monocytes/macrophages-mediated inflammation

It has been shown that PKCδ mediates high-glucose-induced Toll-like receptor 4 (TLR4) expression by stimulating NOX, which were reported to activate NF-κB and facilitate inflammatory cytokine secretion in THP-1 cells and monocytes isolated from healthy volunteers [88]. In monocytes, inhibition of either CD36, TLR2, TLR4, TLR6, or PKCδ prevents ox-LDL-induced PKCδ/IRAK1/JNK1/AP-1 axis activation and IL-1β production [89]. However, Hsu [90] et al. argued that curcumin activates the PKCδ/ERK1/2/HO-1 pathway, which inhibits LPS-induced IL-1 and IL-6 expression in monocytes. Furthermore, PKCδ siRNA administration in diabetic rats resulted in significantly decreased mediators of inflammation in plasma and from macrophages (IL-1, TNF-α, IL-6, MCP-1, KC/IL-8, and PAI -1), indicating a pro-inflammatory role of PKCδ in diabetic atherosclerosis [86].

Except for endothelial NO synthesized by eNOS isoform, NO can also be produced by neuronal NOS (nNOS) and inducible NOS (iNOS) in cells such as macrophages [91]. Inducible NOS in atherosclerotic plaques aggravates the inflammatory process. It has also been demonstrated that ox-LDL-induced iNOS expression in macrophages promotes foam cell formation and plaque development [92]. Genetic deletions of iNOS in hyperlipidemic ApoE^−/−^ mice also resulted in reduced macrophages infiltration, foam cell formation, and decreased lesion size, indicating iNOS’s pro-atherogenic role [93, 94]. Leppänen et al. [95] pointed out that inhibition of PKCδ suppressed iNOS and NO generation via IRF1 inhibition in macrophages. Wu et al. [96] also reported that PKCδ was involved in AGE-induced iNOS expression in RAW 264.7 macrophages. In addition, while Hua et al. [97] found no significant difference in NO production between high-glucose- and normal glucose-cultured RAW 264.7 macrophages, they found higher LPS-induced NO generation, iNOS expression, and interleukin-1 beta (IL-1b) secretion in HG-cultured cells, which is partly mediated by PKCδ/p38 MAPK/NF-κB pathway. Additionally, Bhatt et al. [98] observed that peptidoglycan (PGN) enhanced iNOS expression and NO production through PKCδ/NF-κB pathway activation.

### PKCδ regulates monocytes adhesion, infiltration, and differentiation

In response to chemokines such as MCP-1, monocytes migrate and adhere to activated endothelial cells [99]. Several adhesion molecules, including P-selectin, E-selectin, very late antigen-4 (VLA-4), VCAM-1, and ICAM-1, are involved in the monocytes–endothelial cells interaction [100]. Then monocytes infiltrate into the subendothelial space (diapedesis) and differentiate into macrophages [99]. CD11b, TLR-4, and CD14 are classical markers of M1 macrophages, while p21 and MMP9 facilitate M2 polarization [101–103]. Curcumin, demethoxycurcumin (DMC), and bisdemethoxycurcumin (BDMC), which are the major active components of curcuminoids, suppressed matrix invasion during PMA-induced THP-1 differentiation [104]. The mechanism involves inhibition of the PKCδ/NADPH oxidase/ROS pathway and subsequently CD11 b and MMP 9 expression. Tsai et al. [105] reported that overexpression of thrombomodulin (TM) enhanced macrophage markers CD 14 and CD 68 in PMA-induced THP-1, while inhibition of TM by siRNA suppressed PMA-induced p21^Cip1/WAF1^ expression via ERK1/2-NF-kB p65 signaling. However, PMA-induced p21Cip1/WAF1 expression, CD14-positive cell labeling intensity, and ERK1/2 phosphorylation were significantly reduced when PKCδ was knocked down. Notably, PKCδ was found to be highly expressed in human atherosclerotic arteries and colocalized with TM in CD68-positive infiltrated macrophages of plaques, indicating a coordinating relationship between TM and PKCδ in plaque formation. In addition, calcium dobesilate reduces CD14, TLR4, and MMP9 expression during monocyte-to-macrophage differentiation, which is mediated by the PKCδ/NADPH oxidase/MAPK/NF-κB signaling pathway [106].

### PKCδ regulates cholesterol uptake and foam cells formation

Foam cell formation is a crucial process in the initiation and progression of atherosclerotic plaque formation. Monocytes-derived macrophages uptake modifies LDL in two ways: receptor-dependent pinocytosis and receptor-independent endocytosis [100]. Then cholesterol undergoes esterification and accumulates in macrophages, leading to foam cell formation. Chen et al. [107] reported PKCδ translocation in the early phase of lipid accumulation in oleic acid (OA)-induced RAW264.7 macrophages. Ma et al. [108] also demonstrated that PKCδ mediates cholesterol accumulation in PMA-activated macrophages. Scavenger receptor class A (SR-A) and CD36 play vital roles in receptor-dependent endocytosis of lipoprotein-derived cholesterol. Inhibition of PKCδ resulted in decreased expression of SR-A and CD36 via PI3K/Akt and ERK inhibition, which inhibited oxidized LDL (OxLDL) uptake and intracellular cholesterol accumulation in both THP-1-derived and primary macrophages [109]. Notably, PKCδ, phosphorylated ERK, Akt, and SR-A were highly expressed in human atherosclerotic arteries and CD68-positive macrophages, as visualized by immunohistochemical staining. Yakubenko et al. [110] also reported the involvement of Stat3/PKCδ/p38MAPK in IL-13-induced CD36 expression in monocytes/macrophages, which is inhibited by α_M_β_2_ integrin activation or clustering. However, Szilagyi et al. [87] analyzed the effects of PKCδ inhibition on human monocytic cell lines and primary human monocytes, and did not find a detectable effect on oxLDL uptake and foam cell formation. The same result was also found in bone marrow-derived macrophages from PKCδ knockout mice and macrophages isolated from patients with rare null mutations in the PRKCD gene.

Furthermore, abnormal HDL metabolism in patients with diabetes increases the risk of atherosclerosis [111]. ABCA1 and ABCG1 mediate the efflux of cholesterol and phospholipids from macrophages to HDL, providing protection against plaque formation. Diabetes increases the level of unsaturated fatty acids (uFAs), which was shown to destabilize ABCA1 protein in murine macrophages and impair the ABCA1 pathway through a PKCδ-dependent pathway [111]. However, Ku et al. [112] argued that PKCδ may act oppositely. Depletion of PKCδ reduced ABCA1 and ABCG1 proteins and did not reverse the repressive effect of unsaturated fatty acids.

### PKCδ regulates macrophages apoptosis

Vogl et al. [113] reported a pro-apoptotic role of PKCδ in oxidized phospholipid-induced apoptosis of RAW264.7 macrophages. Li et al. [85] studied mice with selective knockout of PKCδ in macrophages fed with an atherogenic diet (AD) and a very high-fat diet (HFD). They reported that PKCδ KO/ApoE-/- mice showed accelerated aortic atherosclerotic lesions compared with ApoE-/- mice fed with either AD or HFD. Moreover, both AD and HFD led to increases in the number of macrophages in aortic plaques and spleen in PKCδ KO/ApoE-/- mice compared with ApoE-/- mice due to decreased apoptosis and increased proliferation but not increased monocytes uptake. The mechanism involves PKCδ-induced inhibition of P85/PI3K and subsequent elevated phosphorylation levels of pro-survival cell signaling proteins Akt and FoxO3a, and reduced pro-apoptotic protein Bim.

## PKCδ regulates other pathophysiologic processes in diabetic atherosclerosis

Observational studies have demonstrated that high levels of LDL, apolipoprotein B (apo B) and triglycerides increase the risk of atherosclerosis, whereas high levels of HDL and apolipoprotein A (apo A) are associated with a lower risk of atherosclerosis [54]. Several studies have reported on the modulation of lipid metabolism by PKCδ. Bezy et al. found that higher levels of PKCδ in obese individuals were positively correlated with fasting glucose and circulating triglycerides. Overall or liver-specific PKCδ inhibition enhanced hepatic insulin signaling and downregulated the expression of gluconeogenic and lipogenic enzymes [114]. The hepatic low-density lipoprotein receptor (LDLR) removes LDL from the blood, thereby slowing the atherosclerotic process [115]. PKCδ was shown to upregulate hepatic LDLR protein levels. However, Choi et al. argued the inhibition of PKCδ by rottlerin seems to have no effect on LDLR expression as well as Apo B expression [116]. Berberine is a compound isolated from a Chinese herb that has been shown to lower serum cholesterol, triglycerides and LDL [117]. It also increases the expression of ABCA1 protein in hepatocytes by inhibiting PKCδ. ABCA1 mediates the transport of cholesterol and phospholipids from cells to Apo A-I to generate nascent HDL particles [118]. Notably, Apo A-1 expression was significantly upregulated in PKCδ^−/−^ hearts [119].

In addition to ECs, VMSCs and monocytes/macrophages, other cell types such as dendritic cells (DCs), T cells and B cells are involved in the atherosclerotic process [120]. Similar to monocytes, DCs and T cells are attracted to the intima by endothelial adhesion molecules and chemokines. DCs take up LDL components and activate adaptive immunity. T cells, especially CD4 + T cells, are activated at the site of the lesion and produce pro-atherosclerotic mediators. B cells are occasionally present at the site of the lesion but accumulate on the abluminal and adventitial side of the atheroma. Interestingly, B cells are thought to play a protective role. Miyamoto [121] et al. studied bone marrow cells from systemic PKCδ knockout mice. They found an increase in circulating B cells and no change in other myeloid cells. However, the increase in B cells may lead to enhanced autoimmunity and lymphoproliferative syndrome [121–123]. Hamdorf et al. reported that PKCδ is a key mediator in the differentiation of hematopoietic stem cells to myeloid DCs [124]. In addition, PKCδ mediates antigenic macrophagocytosis of DCs and promotes the secretion of T-cell stimulatory cytokines, which are essential for T-cell activation [125–127]. Interestingly, PKCδ also plays a role in regulating apoptosis of mature DCs and T cells, thus preventing persistent immune activation [128, 129]. In addition, PKCδ is involved in the migration of T lymphocytes in the bloodstream, which is essential for the immune response [130]. However, these studies were conducted in the absence of diabetic atherosclerosis and further research is needed.

## PKCδ inhibitors and their applications

PKCδ is activated in several pathophysiological processes of atherosclerosis, and it is a potent therapeutic target for diabetic atherosclerosis. Several PKCδ pathway inhibitors have been documented. We describe these inhibitors and their applications below and in Table 4.

**Table 4:** PKCδ inhibitors and their applications

PKCδ inhibitor	Disease model	Pathophysiological processes
Rottlerin	Atherosclerosis	Increases NO production, decreases ET-1 production in ECs [21, 131]
Rottlerin	Atherosclerosis	Suppresses ICAM-1 and VCAM-1 expression in ECs [37, 38]
Rottlerin	Atherosclerosis	attenuates the migration and adhesion of neutrophils [35]
Rottlerin	Atherosclerosis	Inhibits NOX expression and ROS production in ECs [42]
Rottlerin	Atherosclerosis	Attenuates apoptosis and senescence of ECs [50, 53]
Rottlerin	Atherosclerosis	Alleviates migration and proliferation of VSMCs [66, 68, 70, 71]
Rottlerin	Atherosclerosis	Inhibits apoptosis of VSMCs [80, 81]
Rottlerin	Atherosclerosis	Suppresses IL-1β and iNOS expression, NO generation in monocytes [89, 95, 97]
Rottlerin	Atherosclerosis	Inhibits SR-A and CD36 in macrophages [109]
Rottlerin	Atherosclerosis	Regulates ABCA-1 and ABCG-1 expression in macrophages [111, 112]
Rottlerin	Diabetic retinopathy	Reverses abnormally increased vascular permeability 30
Rottlerin	Inflammatory lung disorders	Reverses abnormally increased vascular permeability 33
δV1-1	Sepsis-induced vascular damage	Reduces TNF-α-mediated ECs hyperpermeability [18]
δV1-1	Sepsis-induced vascular damage	Reduces neutrophils adhesion and migration [18]
δV1-1	Sepsis-induced lung injury	Attenuates inflammatory cell infiltration and endothelial ICAM-1 and VCAM-1 expression [15]
δV1-1	Inflammation-induced tissue damage	Inhibits neutrophils adhesion and migration [17]
Polydatin	Atherosclerosis	Attenuates H_2_O_2_-induced oxidative stress in ECs [44]
Curcuminoids	Atherosclerosis	Suppresses matrix invasion during PMA-induced THP-1 differentiation [104]
Calcium dobesilate	Diabetic retinopathy	Reduces vascular leakage [132]
Calcium dobesilate	Atherosclerosis	Attenuates monocyte-to-macrophage differentiation [106]

### Rottlerin

Rottlerin, also known as mallotoxin, is a natural chemical extracted from *Mallotus philippinensis* and is one of the most frequently used PKCδ inhibitors. It has been shown to regulate several pathophysiologic processes in atherosclerotic plaque formation.

First, rottlerin regulates endothelial cells dysfunction. It increases cytoplasmic free calcium, stimulates NO production, and downregulates ET-1 levels, promoting endothelium-dependent vasodilation [131]. However, Motley et al. [21] noted that rottlerin attenuates the phosphorylation of eNOS and NO production induced by thrombin in HUVECs. It also suppresses diabetes-related enhancement of EP3 receptor-mediated vasoconstriction [14], and was found to reverse the abnormally increased vascular permeability in an experimental model of diabetic retinopathy and inflammatory lung disorders [19, 34]. Characterized by the overexpression of several factors, including ICAM-1, VCAM-1, E-selectin, and others, inflammation is at the core of plaque formation. Rottlerin was also found to suppress thrombin-induced ICAM-1 and VCAM-1 expression in HUVECs [37, 38], and to inhibit the phosphorylation of MARCKS and attenuate the migration and adhesion of neutrophils [35]. Moreover, rottlerin completely reversed AGE-induced NOX activation and ROS generation in human aortic endothelial cells, indicating an antioxidative role [42]. In addition, inhibition of the PKC δ/p53 pathway by rottlerin attenuates H_2_O_2_-induced bovine aortic endothelial cells apoptosis, and rottlerin regulates endothelial senescence by regulating PKC δ-mediated NO production [50, 53].

Second, rottlerin regulates smooth muscle cells dysfunction. Although the role of PKCδ in regulating VSMCs migration and proliferation is still under debate, evidence supports PKCδ as a promoter. Rottlerin has been shown to inhibit COX-2 expression and PDGF-induced ERK1/2 activation, thus alleviating VSMCs migration and proliferation [66, 68]. Moreover, rottlerin was reported to restrain HG-induced TNF-α procession and TNF-α autocrine-activated PKCδ, in turn decelerating VSMCs growth [70, 71]. In regulating VSMCs apoptosis, rottlerin inhibits the accumulation and phosphorylation of p53 and caspase 3-mediated PKCδ cleavage, indicating an anti-apoptotic role [80, 81].

Third, rottlerin regulates monocytes/macrophages dysfunction. It abrogates Ox-LDL-induced IL-1β expression and inhibits iNOS expression and NO generation in monocytes, providing an anti-inflammatory function [89, 95, 97]. Rottlerin also attenuates Ox-LDL uptake by inhibiting the expression of scavenger receptors, including SR-A and CD36, in macrophages [109]. Unsaturated fatty acids (uFAs) were reported to reduce ABCA1 and ABCG1 activity, and rottlerin was reported to abolish the effects of uFAs [111]. However, Ku et al. [112] reported that neither rottlerin nor PKCδ siRNA alleviated uFA-reduced ABCA1 and ABCG1 expression.

### δV1-1

δV1-1 is a nontoxic peptide antagonist that selectively targets PKC δ. It consists of a peptide derived from the first unique region (V1) of PKC δ (SFNSYELGSL; amino acids 8 to 17) coupled via an N-terminal Cys-Cys bond to a membrane-permeant peptide sequence in the HIV TAT gene product (YGRKKRRQRRR; amino acids 47–57 of TAT). It was shown that δV1-1 reduced TNF-α-mediated hyperpermeability as well as neutrophils adhesion and migration across HBMVECs in sepsis-induced vascular damage [18]. In vivo studies also showed that δV1-1 attenuated inflammatory cell infiltration and endothelial ICAM-1 and VCAM-1 expression [15]. It also inhibited the adhesion and migration of bone marrow neutrophils (BMNs) under low shear and near bifurcations [17]. However, Ahn et al. [16] reported a deleterious role of the inhibitor in regulating permeability and neutrophils migration in mice with acute lung injury.

### Others

Polydatin, extracted from the root stem of a traditional Chinese herbal medicine, *Polygonum cuspidatum Sieb*, was reported to be an antioxidant involved in antiplatelet aggregation and antiatherosclerosis [44]. It is an effective inhibitor of the PKC δ pathway and attenuates H_2_O_2_-induced oxidative stress injury in HUVECs [44]. Curcumin, demethoxycurcumin (DMC), and bisdemethoxycurcumin (BDMC), isolated from the rhizomes of *Curcuma longa*, are the major active components of curcuminoids and exhibit anti-inflammatory, anticarcinogenic, and anti-atherosclerotic biological effects. They were found to suppress matrix invasion during PMA-induced THP-1 differentiation by inhibiting PKCδ/NOX/ROS and subsequent CD11 b and MMP 9 expression [104]. Calcium dobesilate (CaD) is an angioprotective drug mainly used for the treatment of diabetic retinopathy and chronic venous insufficiency. It was shown to reduce vascular leakage via PKCδ inhibition in an experimental model of diabetic retinopathy [132]. During monocyte-to-macrophage differentiation, it also attenuates the PKCδ/NOX/MAPK/NK-κB pathway and the expression of differentiation markers [106].

## The comparison of functions between PKCδ and other PKCs in diabetic atherosclerosis

PKC is a family of serine/threonine-associated protein kinases that consists of three subfamilies: classical PKC, novel PKC, and atypical PKC. The activity of classical PKC (α, βI, βII, and γ) is dependent on DAG and calcium. The activity of novel PKC (δ, ε, θ, and η) is dependent on DAG but not on calcium. In contrast, atypical PKC (ζ, ι/λ) are dependent on both DAG and calcium [2]. All PKC isozymes play a unique role in the development of atherosclerosis [6]. However, in diabetic atherosclerosis research, PKCα, PKCβ and PKCδ are the predominantly studied targets. PKCα has been reported to induce endothelial hyperpermeability, downregulate vascular smooth muscle cells apoptosis, and exacerbate monocytes/macrophages-mediated inflammation responses [83, 88, 97, 133, 134]. More studies have focused on the role of PKCβ in diabetic atherosclerosis. It has been documented as a promoter of endothelial cells, vascular smooth muscle cells and monocytes/macrophages dysfunction [30, 72, 83, 134–139]. The functions of these PKC isoforms in diabetic atherosclerosis have been listed in Table 5. Although PKCα, PKCβ and PKCδ have been reported to mediate various pathophysiological processes in the development of diabetic atherosclerosis, they have similarities. More studies on the different roles of PKC isoforms in diabetic atherosclerosis are needed.

**Table 5:** The role of PKC isoforms in diabetic atherosclerosis

PKC isoforms	Working model	Pathophysiological processes
PKCα	PAECs	Increases endothelial permeability [133]
PKCα	A7r5 rat aortic VSMCs, human umbilical artery VSMCs, human aortic VSMCs	Inhibits serum withdrawal-induced VSMCs apoptosis [83]
PKCα	RAW 264.7, THP-1	Increases monocytes/macrophages-mediated inflammation [88, 97, 134]
PKCβ	Diabetic patients, capillary BRECs, BRPs	Increases vasoconstriction [30] Inhibits vasodilation [135]
PKCβ	BAECs, HAECs, HUVECs	Increases endothelial VCAM-1 and ICAM-1 expression, enhances plaque formation, complexity, and cholesterol content [137]
PKCβ	Human VSMCs	Mediates thrombin-stimulated VSMCs migration [72]
PKCβ	A7r5 rat aortic VSMCs, human umbilical artery VSMCs, human aortic VSMCs	Inhibits serum withdrawal-induced VSMCs apoptosis [83]
PKCβ	THP-1 cells, Raw 264.7, U937, diabetic ApoE-null mice	Increases monocytes/macrophages-mediated inflammation [134, 136, 137, 139]
PKCβ	Diabetic rats	Increases macrophages recruitment and ICAM-1 and MCP-1 protein expression in the kidney [138]
PKCδ	Capillary BRECs, BRPs, HUVECs, diabetic rats	Increases vasoconstriction [14, 29, 30] Inhibits vasodilation [13]
PKCδ	HRMECs, diabetic mice	Increases endothelial permeability [19]
PKCδ	HAECs, ECV 304, aortic segments from old mice	Mediates NADPH oxidase-dependent oxidant stress [42]
PKCδ	HUVECs	Increases endothelial apoptosis
PKCδ	Rat aortic VSMCs, human VSMCs	Promotes VSMCs migration and proliferation
PKCδ	Human VSMCs	Mediates thrombin-stimulated VSMCs migration [72]
PKCδ	RAW 264.7, THP-1, diabetic rats	Increases monocytes/macrophages-mediated inflammation [86, 88, 97]
PKCδ	Diabetic ApoE-null mice	Increases macrophages apoptosis and decreases their proliferation, alleviates plaque progression and splenomegaly [85]
PKCδ	Tsc1- null MEFs	Increases LDLR expression [115]

*PAECs* Porcine aortic endothelial cells, *BRECs* Bovine retinal endothelial cells, *BRPs* Bovine retinal pericytes, *BAECs* Bovine aortic endothelial cells, *HAECs* Human aortic endothelial cells, *HUVECs* Human umbilical vein endothelial cells, *ICAM-1* Intercellular cell adhesion molecule-1, *VCAM-1* Vascular cell adhesion molecule-1, *MCP-1* Monocyte chemoattractant protein-1, *HRMECs* Human retina microvascular endothelial cells, *NADPH* Nicotinamide adenine dinucleotide phosphate, *MEFs* Mouse embryonic fibroblasts, *LDLR* Low density lipoprotein receptor

## Summary

Diabetes has long been recognized as a pro-atherosclerotic process, but the mechanisms of diabetic atherosclerosis have not been fully elucidated. The pathophysiology of diabetic complications involves the activation of the polyol pathway, nonenzymatic glycation, the advanced glycation end products (AGEs) pathway, elevated reactive oxygen (ROS) production, and activation of the diacylglycerol (DAG)-protein kinase C (PKC) pathway. In this review, we focused on the role of PKCδ, a PKC isoform, in regulating the function of endothelial cells (Fig. 2), vascular smooth muscle cells (Fig. 3), and monocytes/macrophages (Fig. 4) in the process of atherosclerotic plaque formation.

In non-DM VSMCs studies, the prevailing idea is that PKCδ is a promoter of VSMCs proliferation, migration, and apoptosis. However, Liu et al. [58] argued that both overexpression and knockout of PKCδ suppress the proliferation and migration of VSMCs, indicating a dual role of PKCδ in regulating VSMCs proliferation and migration. In DM VSMCs studies, current evidence supports PKCδ as an enhancer of VSMCs proliferation and migration but not apoptosis. In some non-DM ECs studies, PKCδ was reported to be involved in the production of vasodilators such as NO, prostacyclin, and antioxidants and to contribute to endothelial hyperpermeability and senescence. However, DM studies and most non-DM studies have demonstrated that PKCδ impairs endothelium-dependent vasodilation, exacerbates endothelial hyperpermeability, and leads to endothelial senescence and apoptosis. Furthermore, PKCδ promotes the expression of endothelial adhesion molecules and facilitates leukocytes adhesion and transmigration, which aggravates inflammation and endothelial dysfunction. In monocytes/macrophages studies, PKCδ was reported to play a positive role in myocyte migration, infiltration, and differentiation, inducible NO generation, and macrophages apoptosis. However, the role of PKCδ in regulating foam cell formation is still under debate. Some believe that PKCδ augments scavenger receptor expression, impairs cholesterol efflux, and promotes foam cell formation, while others find no protective effect of PKCδ depletion. However, these three types of cells are not completely isolated. Ren et al. [140] showed that VSMCs promote re-endothelialization in a PKCδ-dependent paracrine mechanism, likely through CXCL7-mediated recruitment of endothelial cells from uninjured endothelium. PKCδ also mediates the transformation of endothelial cells into smooth muscle cell-like cells. Furthermore, PKCδ regulates endothelial NO generation, which influences VSMCs proliferation, migration, and constriction and macrophages polarization [141–144]. Matesanz et al. [145] reported that the inhibition of PKCδ in endothelial cells inhibits VCAM-1 expression and monocytes binding. PKCδ has been regarded as a potential target to alleviate the progression of atherosclerosis. Although in vitro and animal studies have revealed that PKCδ inhibitors, including rottlerin, siRNA and δV1-1, curcumin, polydatin, and calcium dobesilate, restrain several pathophysiologic processes of plaque formation, there is a paucity of clinical evidence. Therefore, more clinical studies are urgently needed to test the effectiveness of PKCδ inhibitors in delaying, stopping, or even reversing atherosclerosis.

## Perspectives

In this review, we discussed the role of PKCδ in regulating several pathophysiologic changes of VSMCs, ECs, and monocytes/macrophages in the process of atherosclerotic plaque formation under DM and non-DM conditions. However, both upregulation and downregulation of PKCδ can lead to similar effects (Supplementary table 1). We reviewed literatures focusing on the function of PKCδ in PKCδ-overexpressed mice but found little evidence. Mice with liver-specific overexpression of PKCδ showed decreased insulin signaling, enhanced lipogenic gene expression, and hepatosteatosis [114]. Epidermis-specific overexpression of PKCδ inhibited skin tumor formation [146]. Thus, more high-quality evidence from PKCδ downregulated or overexpressed DM animals is required. In addition, atherosclerosis is a complex inflammatory disease that involves not just those three types of cells, but also lymphocytes, NK cells, dendritic cells, neutrophils, and others. The importance of these cells in the formation of atherosclerotic lesions and of PKCδ needs to be further explored. Calcium dobesilate is a clinically available drug mainly used in the treatment of diabetic retinopathy and deep venous insufficiency. It was also shown to inhibit monocytes differentiation via PKCδ inhibition, indicating a possible role of calcium dobesilate in the treatment of atherosclerosis. Rottlerin and siRNA, the two most commonly used PKCδ inhibitors, have been proven to alleviate the VSMCs, ECs, and monocytes/macrophages dysfunction. Other natural extracts, such as polydatin and curcumin, have also been proven to protect endothelial cells via PKCδ suppression. Their application in clinical practice is also worth investigating.

### Supplementary Information

**Additional file 1:** **Supplementary table 1.** PKCδ regulates cellular functions under non-DM and DM conditions.

## Data Availability

The datasets used and analyzed during the current study are available from the corresponding author on reasonable request.
