# Supplementary material for: PKCδ regulates the vascular biology in diabetic atherosclerosis
Source: Cell Commun Signal. 2023 Nov 16;21:330. doi: 10.1186/s12964-023-01361-4 (PMC10652453; doi:10.1186/s12964-023-01361-4)
Supplement: Supplementary file 2 — Additional file 1: Supplementary table 1. PKCδ regulates cellular functions under non-DM and DM conditions. [file 12964_2023_1361_MOESM1_ESM.docx]

**Supplementary table 1. PKCδ regulates cellular functions under non-DM and DM conditions**

| **Cellular functions** | **Non-DM** | | | **DM** | | |
| --- | --- | --- | --- | --- | --- | --- |
|  | **control** | **PKCδ inactivation** | **PKCδ activation** | **control** | **PKCδ inactivation** | **PKCδ activation** |
| Endothelial-dependent vasodilation | Base | Decrease[14], [16], [21]  Increase[17]–[19] | Increase[16], [21]  Decrease[17] | Base | Not mention | Not mention |
| Endothelial-dependent vasoconstrictioin | Base | Not mention | Not mention | Base | Decrease[23], [24] | Increase[23] |
| Hyperpermeability | Base | Increase[26], [27], [33]  Decrease[29]–[31] | Decrease[27] | Base | Increase[28] | Not mention |
| Leukocytes adhesion and migration | Base | Decrease[29], [32], [34], [35]  Increase[33] | Not mention | Base | Not mention | Not mention |
| Endothelial-mediated inflammation | Base | Decrease[21], [37]–[40] | Not mention | Base | Not mention | Not mention |
| Oxidative stress | Base | Decrease[43]  Increase[45] | Not mention | Base | Decrease[42] | Not mention |
| EndoMT | Base | Decrease[47] | Not mention | Base | Not mention | Not mention |
| Endothelial senescence and apoptosis | Base | Decrease[50], [51] | Increase[51] | Base | Decrease[48] | Not mention |
| VSMCs proliferation | Base | Increase[57]  Decrease[58], [59], [62] | Decrease[62] | Base | Decrease[66], [68], [69] | Not mention |
| VSMCs migration | Base | Decrease[58], [59], [62] | Decrease[62] | Base | Decrease[68], [69] | Not mention |
| VSMCs apoptosis | Base | Decrease[57], [73], [74], [78] | Increase[73] | Base | Not mention | Not change[80] |
| Monocytes/macrophages-mediated inflammation | Base | Decrease[86], [93], [96]  Increase[87] | Not mention | Base | Decrease[85], [95] | Not mention |
| Monocytes adhesion, infiltration, and differentiation | Base | Decrease[102]–[104] | Not mention | Base | Not mention | Not mention |
| Cholesterol uptake and foam cell formation | Base | Decrease[107], [110]  Not change[109]  Increase[111] | Not mention | Base | Not mention | Not mention |
| Macrophages apoptosis | Base | Decrease[112] | Not mention | Base | Not mention | Not mention |
| B cells proliferation | Base | Increase[123] | Not mention | Base | Not mention | Not mention |
| B cells death | Base | Not mention | Increase[123] | Base | Not mention | Not mention |
| DCs activation | Base | Decrease[124], [125] | Not mention | Base | Not mention | Not mention |
| Mature DCs apoptosis | Base | Decrease[128] | Not mention | Base | Not mention | Not mention |
| T cells activation | Base | Decrease[125], [127] | Not mention | Base | Not mention | Not mention |
